# Recombinant Factor VIIa: Hemostatic Adjunct in the Coagulopathic Burn Patient

**Published:** 2009-07-01

**Authors:** Jeremiah T. Martin, Fuad Alkhoury, Bryan C. McIntosh, Phillip Fidler, John Schulz

**Affiliations:** Hospital of Saint Raphael, 1450 Chapel St, New Haven, CT 06511

## Abstract

**Introduction:** Recombinant factor VIIa (rFVIIa; NovoSeven) is well recognized as an effective hemostatic agent in the management and prophylaxis of patients with hemophilia. We report here the successful use of rFVIIa in a coagulopathic burn patient. **Methods:** A 63-year-old man was admitted with significant upper-body burns in a total body surface area of 60%. Initial management included early intubation and escharotomies, with subsequent admission to the burn unit. Fascial excision was carried out with allograft placement. During a complicated hospital course, decline in platelet function was noted and was associated with the development of a generalized coagulopathy with elevated international normalized ratio. Following a routine follow-up debridement and autografting, extensive bleeding was noted from donor sites. A period of increasing hemodynamic instability followed in the burn unit, with serial hematocrit measurements pointing toward ongoing bleeding from the surgical sites. Following administration of significant amounts of blood product, it was decided to administer rFVIIa per pharmacy protocol. **Results:** Within 4 hours of administration of rFVIIa, the patient was noted to be hemodynamically stable with unchanging serial hematocrit measurements. Hemostasis was attributed to the use of rFVIIa with prior administration of platelets. **Conclusions:** Our case demonstrates the successful use of rFVIIa in the severely coagulopathic burn patient.

## CASE REPORT

We present the case of a 63-year-old man who was admitted to our hospital with significant upper-body burns. The previous night he had been cooking when his clothing ignited. He did not call for help until the following morning.

Initial assessment revealed approximately 60% total body surface area burn, most of which was full thickness involving his torso and upper extremities. In addition, there was some minor involvement of his lower extremities. The patient complained of pain and inability to pass urine. Limited history was obtained in the emergency department. The patient denied any allergies and was not taking any medications.

Intubation for airway protection was performed early in the trauma room, and the patient was transferred to the burn unit where extensive fasciotomies to bilateral arms, hands, chest, and abdomen were performed at the bedside. Initial laboratory reports revealed acute renal failure with a creatinine level of 2.2, which marked the beginning of an undulating course. The next day, the patient was taken to the operating room for his first of several procedures. Fascial excision of both arms and left flank, with allograft, was performed. Skin biopsy was taken per protocol, with a view to obtaining cultured epidermal autograft.

His hospital course was complicated by pneumonia and polymicrobial sepsis. Organisms isolated from blood and wound-bed cultures included *Pseudomonas*, *Aspergillus*, vancomycin-resistant *Enterococcus*, and *Klebsiella*. Broad-spectrum antibiotic coverage was instituted.

By the sixth week of hospitalization, a decline in platelet function was noted from more than 150 × 10^9^ to less than 15 × 10^9^. This was paralleled by a generalized coagulopathy with a rise in international normalized ratio to more than 1.52. At this time, the patient had been taken to the operating room for fascial excision of the lower back with limited autografting. Although the procedure was uneventful, by the end of the operative procedure, bleeding was noted from the autograft donor sites. This was difficult to control, but sufficient hemostasis was achieved.

Upon return to the burn unit, the patient became increasingly hemodynamically unstable, with serial hematocrit measurements revealing ongoing bleeding despite blood transfusions. After 12 units of packed red blood cells, 16 units of fresh frozen plasma, and 16 units of platelets, consideration was given to recombinant activated factor VII (rFVIIa). This was administered per pharmacy protocol, with subsequent stabilization of the patient and cessation of ongoing blood losses. A dose of 90 µg/kg was chosen on the basis of the use of the agent in other scenarios.

We present this case as a report of successful hemostasis in a patient with severe burn injury after the administration of rFVIIa.

## DISCUSSION

Recombinant factor VIIa (NovoSeven) was initially conceived and is well recognized as an effective hemostatic agent in the management and prophylaxis of patients with hemophilia. Almost identical to human factor VIIa, the recombinant form is genetically engineered in cultured baby hamster kidney cells. An increasing body of evidence is appearing that supports the use of rFVIIa in other situations that require prompt hemostasis.^1^

The traditionally understood coagulation cascade has come under scrutiny, and recent work, most notably that of Hoffman et al,^2^–^4^ has given rise to the “cell-based” model of coagulation. Three stages have been delineated:

1. *Initiation*: Endothelial injury exposes tissue factor (TF)-bearing cells. FVII binds to TF and cleaves FX, which then binds to FVa on the TF-bearing cell surface. Thus, thrombin is cleaved (by the FVa/Xa complex, thrombinase), which proceeds to cleave fibrinogen in addition to activating platelets, stimulating the release of FVIII from von Willebrand factor and activating FIXa. This stage is limited to the area of endothelial damage by circulating tissue factor pathway inhibitors.
2. *Amplification*: Occurring on the surface of activated platelets, the FVa/Xa complex activates more thrombin, increasing fibrin production and the formation of a fibrin clot. These platelets have been activated by the thrombin released in the initiation phase and are characteristic in their expression of surface-binding sites for FVIIa/FXIa complex (tenase) and thrombinase.
3. *Propagation*: The fibrin clot increases in density as more platelets are activated in the region of injury with the amplification of this cascade. As mentioned, tissue factor pathway inhibitor inhibits inappropriate initiation of clot outside the zone of injury. Circulating antithrombin and protein C/S also play important roles in the limitation of clot progression.

In the cell-based model of coagulation, FVIIa serves an important role both in the initiation of coagulation and in the activation of platelets. The administration of higher doses of rFVIIa has revealed that it can also function independently of TF by binding directly to the surface of the activated platelet^3^ and activating FXa there with a burst of fibrin. Again, this coagulation activation is limited to the site of active bleeding both by circulating inhibitors and by the requirement for activated platelets.

Our case demonstrates the use of rFVIIa as a hemostatic adjunct in a patient with severe burn injury and life-threatening hemorrhage after surgery. His coagulopathy arose from several factors including ongoing bleeding with the consumption of factors, systemic sepsis, and uremia secondary to acute renal failure. Within hours of administration of rFVIIa, the patient's hemodynamic profile improved, and serial hematocrit values revealed that repeat transfusions were then no longer required.

It should be noted that prior to the administration of rFVIIa, platelet transfusion was carried out. Case reports have demonstrated a reduction in bleeding time with rFVIIa administration in the setting of thrombocytopenia^4^–^7^; this being explained by the generation of thrombin during the initiation phase by the TF/FVIIa complex. However, the efficacy is clearly greater in the presence of activated platelets, thereby invoking the amplification phase.

Of the off-label indications for rFVIIa, the use in septic patients or patients with burn injury is regarded with some caution because of the risk of inadvertent overactivation of the coagulation cascade with systemic coagulation. Nonetheless, there are case reports of successful application of rFVIIa in critical situations, as illustrated by our patient. Despite the sometimes inadequate efficacy of the therapy in these situations, no thrombotic complications have been observed.^8^

It is important to correct all areas of the coagulation cascade when faced with refractory bleeding. Balanced transfusion therapy^9^ should serve as the initial cornerstone of management. Although the specific components of such an approach are variable, the transfusion of red blood cells should be accompanied with fresh frozen plasma and cryoprecipitate.^10^ In uremic patients, or those at risk of platelet dysfunction, the adjunctive use of desmopressin (DDAVP) should also be considered prior to the use of a novel agent such as rFVIIa.^11^,^12^

Recombinant factor VIIa, coupled with newfound understanding of the coagulation cascade in vivo, has many promising applications in surgical therapy. There is increasing interest, in particular, in the use of this novel agent in the unique setting of patients with burn injury in whom serial excisions and grafting are required, especially vulnerable and compromised patients.^13^,^14^ Further case-controlled trials are necessary to confirm the efficacy, safety, and ideal dosing of this novel drug in patients with burn injury.

## References

[1] Enomoto TM, Thorborg P (2005). Emerging off-label uses for recombinant activated factor VII: grading the evidence. Crit Care Clin.

[2] Hoffman M (2003). Remodeling the blood coagulation cascade. J Thromb Thrombolysis..

[3] Hoffman M (2003). A cell-based model of coagulation and the role of factor VIIa. Blood Rev..

[4] Hoffman M, Monroe DM (2001). A cell-based model of hemostasis. Thromb Haemost.

[5] Conesa V, Navarro-Ruiz A, Borras-Blasco J, Mompel A, Gomez A, Gonzalez M (2005). Recombinant factor VIIa is an effective therapy for abdominal surgery and severe thrombocytopenia: a case report. Int J Hematol.

[6] Goodnough LT (2004). Experiences with recombinant human factor VIIa in patients with thrombocytopenia. Semin Hematol..

[7] Kjalke M, Ezban M, Monroe DM, Hoffman M, Roberts HR, Hedner U (2001). High-dose factor VIIa increases initial thrombin generation and mediates faster platelet activation in thrombocytopenia-like conditions in a cell-based model system. Br J Haematol..

[8] Bianchi A, Jackson D, Maitz P, Thanakrishnan G (2004). Treatment of bleeding with recombinant factor VIIa in a patient with extensive burns. Thromb Haemost..

[9] Stensballe J, Lethagen S, Lippert F, Johansson PI (2005). [Rational treatment of uncontrollable, life-threatening hemorrhage with recombinant factor VIIa]. Ugeskr Laeger..

[10] Johansson PI, Hansen MB, Sorensen H (2005). Transfusion practice in massively bleeding patients: time for a change?. Vox Sang..

[11] Franchini M (2007). The use of desmopressin as a hemostatic agent: a concise review. Am J Hematol..

[12] Hedges SJ, Dehoney SB, Hooper JS, Amanzadeh J, Busti AJ (2007). Evidence-based treatment recommendations for uremic bleeding. Nat Clin Pract Nephrol..

[13] Johansson PI, Eriksen K, Alsbjorn B (2006). Rescue treatment with recombinant factor VIIa is effective in patients with life-threatening bleedings secondary to major wound excision: a report of four cases. J Trauma..

[14] Johansson PI, Eriksen K, Nielsen SL, Rojkjaer R, Alsbjorn B (2007). Recombinant FVIIa decreases perioperative blood transfusion requirement in burn patients undergoing excision and skin grafting—results of a single centre pilot study. Burns..

